# Composite Materials and Films Based on Melanins, Polydopamine, and Other Catecholamine-Based Materials

**DOI:** 10.3390/biomimetics2030012

**Published:** 2017-07-06

**Authors:** Vincent Ball

**Affiliations:** 1 Faculté de Chirurgie Dentaire, Université de Strasbourg, 8 rue Sainte Elisabeth, 67000 Strasbourg, France; vball@unistra.fr; Tel.: +33-03-6885-3384; 2 Unité Mixte de Recherche 1121, Institut National de la Santé et de la Recherche Médicale, 11 rue Humann, 67085 Strasbourg Cedex, France

**Keywords:** polydopamine, melanin, composite films, core-shell nanoparticles

## Abstract

Polydopamine (PDA) is related to eumelanins in its composition and structure. These pigments allow the design, inspired by natural materials, of composite nanoparticles and films for applications in the field of energy conversion and the design of biomaterials. This short review summarizes the main advances in the design of PDA-based composites with inorganic and organic materials.

## 1. Introduction

The strong underwater adhesion of mussels [1], and the exceptional mechanical properties of squid beaks [2] and see worm jaws [3], which are achieved without high inorganic content contrarily to bones and teeth where the inorganic material content is high, raised the question how such exceptional properties are reached. Analysis of the composition and structure of the mussel byssus, squid beaks, and marine hydroid perisarc [4] showed that those materials are essentially composites between proteins, polysaccharides, and melanin-related materials. The chemical principles implied in the formation of those composites rely on the rich chemistry of catechol and catecholamines, the molecular building blocks of melanins: pH-dependent redox chemistry, electrostatic interactions, and complexation with metallic cations are implied in the formation of melanins, their interaction with proteins and other materials [5,6]. The need for simplified design principles of adhesives mimicking the properties of the mussel foot proteins (mfps) led to the use of dopamine as the precursor of films adhering to the surface of almost all known materials [7]. Indeed, dopamine contains a catechol moiety (like the l-3,4-dihydroxyphenylalanine (l-DOPA) residue in mfps) and a primary amine function (like l-lysine in mfps). The films obtained during the oxidation of dopamine using either O_2_ dissolved in water (at basic pH values) or exogeneous oxidants (NaIO_4_, sodium peroxodisulfate) [8] offer fascinating opportunities not only to coat all kinds of materials (metals, oxides, polymers), but also to post-functionalize them with either inorganic or organic compounds. During all synthesis routes of polydopamine (PDA) and related materials, the catecholamine solubilized in an appropriate buffer solution has simply to be mixed with an excess of oxidant to yield to the formation of particles/precipitates in solution and to a homogeneous film at interfaces after an initial island growth regime. Note that the growth rate of the PDA films and their composition depends markedly on the used buffer and the used oxidant [8,9,10].

The strong adhesion afforded by those PDA, and related catechol- and catecholamine-based coatings (norepinephrine [9], l-DOPA), as well as their broad reactivity with cations and nucleophiles (thiols and amines), offers a broad range of applications, allowing the design of new composite materials, particles, and films [10,11,12]. One of the driving principles in the manipulation of PDA is to consider its close compositional and structural analogy with eumelanins [13,14], the brown-black dye affording photoprotection to the skin [15].

It is the aim of this short review to summarize the main recent advances in the design of PDA-, melanin-, and catechol-based composite materials, particles, and films. The main principles in the design of those composites will be explained without trying to be exhaustive, but rather illustrative. We will, hence, classify the composites obtained with inorganic and organic materials rather than focusing on their possible applications. The rationale behind this classification is that the composites with inorganic and organic materials rely on the different interaction modes afforded by PDA, melanins, or catechols. This mini-review will end with a section devoted to highlighting perspectives in this field.

## 2. Composites of Polydopamine and Inorganic Materials

### 2.1. Carbon-Based Composites

Graphene oxide can be reduced in graphene in the presence of norepinephrine which, in turn, is oxidized and polymerized in poly(norepinephrine). The resulting material covers graphene and affords colloidal stability to the composite [16]. When dopamine is modified with an azide function, the PDA-covered graphene can be further functionalized with molecules carrying alkynes through 1,3-dipolar cycloadditions (i.e., via “click chemistry”) [17].

Graphene oxide functionalized with PDA is an efficient support to fix S and CS_2_ in the design of cathodes for Li–S batteries. In this case the volume expansion of sulphur during discharge, and the leaching of sulphur-containing species is strongly limited and allows to increase the number of charge–discharge cycles of the batteries [18].

Carbon nanotubes coated with PDA can be modified with an initiator of atom transfer radical polymerization to coat the nanotubes with polydimethylamino-ethyl methacrylate brushes, which can be subsequently quaternized with CH_3_I. Finally, Pd nanoparticles can be deposited on that composite structure which displayed an excellent electrochemical behavior when deposited on indium tin oxide electrodes [19].

The pyrolysis of PDA at temperatures above 500 °C yields a nitrogen-doped graphitic material because O is lost during the pyrolysis [20]. This concept was used to improve the electrochemical performance of spray-dried Si/graphite (Si/G) composite anodes in Li batteries [21]. The Si and carbon nanopowders were blended and reacted with dopamine (at pH = 8.5 during 24 h using O_2_ as the oxidant) resulting in a PDA-coated blend which was subsequently pyrolyzed to yield a composite displaying an electronic conductivity of 1.44 S m^−1^ compared to 1.2 S m^−1^ for the Si/G blend. More interesting, the capacity retention is of 82% after 100 lithiation–deliathiation cycles for the Si/G-PDA composite, whereas it falls to 6.8% for the uncoated Si/G blend [21].

**Figure 1:**
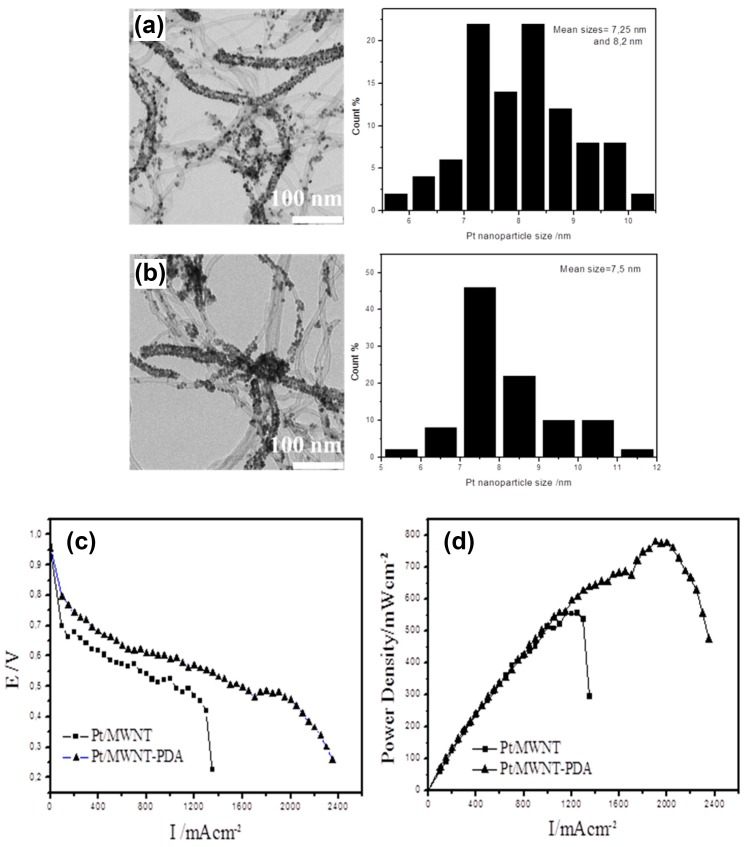
Transmission electron microscopy (TEM) images and corresponding particle size distribution histograms of (**a**) Pt-decorated multi-walled carbon nanotubes (Pt/MWNTs) and (**b**) Pt/MWNTs with polydopamine (Pt/MWNTs-PDA) catalysts used in the design of polyelectrolyte membrane-based fuel cells. (**c**) Polarization and (**d**) power density curves of fuel cell (Pt/MWNTs)_50_ and (Pt/MWNTs-PDA)_50_. The composite membranes were produced by 50 spray cycles on a hot substrate. Reprinted from [22], Copyright (2016), with permission from Elsevier.

Multi-walled carbon nanotubes (MWNT) decorated with PDA are excellent supports for Pt nanoparticles allowing for an optimal Pt utilization (6051 mW mg^−1^ of catalyst) and a high-power density in polymer electrolyte membrane fuel cells (Figure 1). In such a membrane design, PDA plays the same role as Nafion^®^ (DuPont) in other cells to afford a high protonic conductivity, between the anode (oxidation of H_2_) and the cathode (reduction of O_2_). In addition, PDA was shown to protect the electronic conductor, the MWNTs, against corrosion in the hard operation condition of the fuel cell [22].

### 2.2. Composites with Ions and Nanoparticles

The strong interactions between melanins and Na^+^ cations has been used to evaluate melanin–Na^+^ composites as anodes in fuel cells with a λ-MnO_2_ cathode [23].

PDA films deposited on planar surfaces contain enough catechol groups to reduce metallic cations into nanoparticles [7]. In particular cotton coated with PDA was exposed to a solution containing Ag^+^ cations to deposit silver nanoparticles affording excellent antibacterial activity to the fabric [24]. X ray photoelectron spectroscopy has allowed to show that the reduction of Ag^+^ cations into Ag is accompanied by an oxidation of catechol groups into quinones but without affecting the concentration of free radicals in the PDA film [25].

Coating of superparamagnetic Fe_3_O_4_ nanoparticles of different core size with PDA allowed to increase the biocompatibility of those core-shell nanoparticles [26]. The deposition of melanin–Fe_3_O_4_ composite films on Au(111) electrodes allows to catalyse the electroreduction of hydrogen peroxide in alkaline and neutral solutions [27]. 

Citrate-capped gold nanoparticles are useful as localized surface plasmon resonance (SPR) elements and are hence useful for label-free biosensing. Indeed, a binding event causes a change in the refractive index at the surface of the particle and a concomitant red shift in the plasmonic absorbtion bands. When used in solution, separation–redispersion steps are required to separate the particles from the unbound ligands. These separation–redispersion steps induce some particle loss and aggregation. In the latter case, the sensitivity of the aggregates to further changes in the refractive index are lost, as well as their sensing ability. To overcome these problems, and to ensure multiple uses of these sensing elements, their immobilization on transparent surfaces is helpful, provided the particles do not desorb or move on the substrate to finish in a cluster. Such an aggregation process would result in a loss of active surface area. The deposition of a thin PDA film on the surface of citrate-capped Au nanoparticles was found not to hinder the responsiveness of the nanoparticles and to obtain a stable SPR spectrum after several washing–drying cycles which are relevant for real world sensing applications [28].

Core-shell magnetite–polystyrene particles 90 nm in diameter can be aligned to form chains in a magnetic field. When dopamine is oxidized in the presence of this magnetic field the nanoparticles remain aligned even after removing the external magnet. Stable nanochains of up to 20 µm in length can be obtained this way. They can subsequently be aligned in an external magnetic field and used as nanostirrers. The PDA shell can be used to reduce metal cations into metallic nanoparticles affording some catalytic activity to the nanostirrers. These catalysts can be easily separated from the reaction mixture and allow for self-mixing. For instance, Au-decorated PDA-capped nanochains allow the catalysis of the transformation of 4-nitrophenol into 4-aminophenol in the presence of NaBH_4_. When self-stirred in the presence of an external magnetic field, the rate constant of the reaction is of 0.208 min^−1^ in comparison to 0.132 min^−1^ in the absence of a magnetic field. Finally, the PDA-capped nanochains can be easily functionalized with thiol-modified poly(ethylene glycol) (PEG) or with thiol-modified DNA aptamers [29]. 

TiO_2_ nanoparticles were coated with a catechol-based bifunctional initiator of poly(methyl methacrylate) to synthetize inorganic core-organic shell stable particles [30].

### 2.3. Composites with Clays and Zeolites

When l-DOPA is oxidized in the presence of laponite, its polymerization is considerably accelerated with respect to homogeneous l-DOPA solutions, the clay is delaminated and embedded in a gel [31]. Later on, the influence of another clay, saponite, on the formation of a eumelanin-like composite material was investigated [32].

3-(3,4-Dihydroxyphenyl)-dl-alanine (dl-DOPA) was reacted with V_2_O_5_·nH_2_O gels to form a dark blue metallic-colored film after solvent casting of the dl-DOPA + V_2_O_5_·nH_2_O mixture. The lamellar structure of V_2_O_5_ is preserved upon the intercalation of the melanin like material [33], but with an increase in the interlayer spacing. This allowed an increase in the reproducibility of the Li^+^ insertion–deinsertion process in the V_2_O_5_-based gel. In addition, electron paramagnetic resonance spectroscopy showed that the incorporation of the melanin-like material in V_2_O_5_ was accompanied by a partial reduction of V^+5^ cations into V^+4^ cations. Finally, the room temperature electrical conductivity of the composite was increased from 1.1 × 10^−4^ S cm^−1^ to 5.2 × 10^−3^ S cm^−1^ upon incorporation of about 2% (*w*/*w*) of melanin-like material [33].

When 5,6-dihydroxyindole (DHI) and its *N*-methyl derivative are oxidized in the nanopores of zeolite L at a ratio of 10 mg indole derivative to 300 mg porous material, the composite is red. Its dissolution with HF (to remove the inorganic core) and the analysis of the organic content reveals the presence of indole dimers. Hence, the self-assembly process of DHI is hindered in the nanoporous environment of the zeolite. However, when the same oxidation process is performed in the larger pores of SBA-15, a black eumelanin derivative is obtained [34].

## 3. Composites of Polydopamine with Organic Materials

### 3.1. Interactions between Melanins and Porphyrins

Melanins are known to interact strongly with cationic porphyrins to change the optical properties of the dye, in particular its delayed luminescence is substantially reduced in the presence of melanin [35].

**Figure 2:**
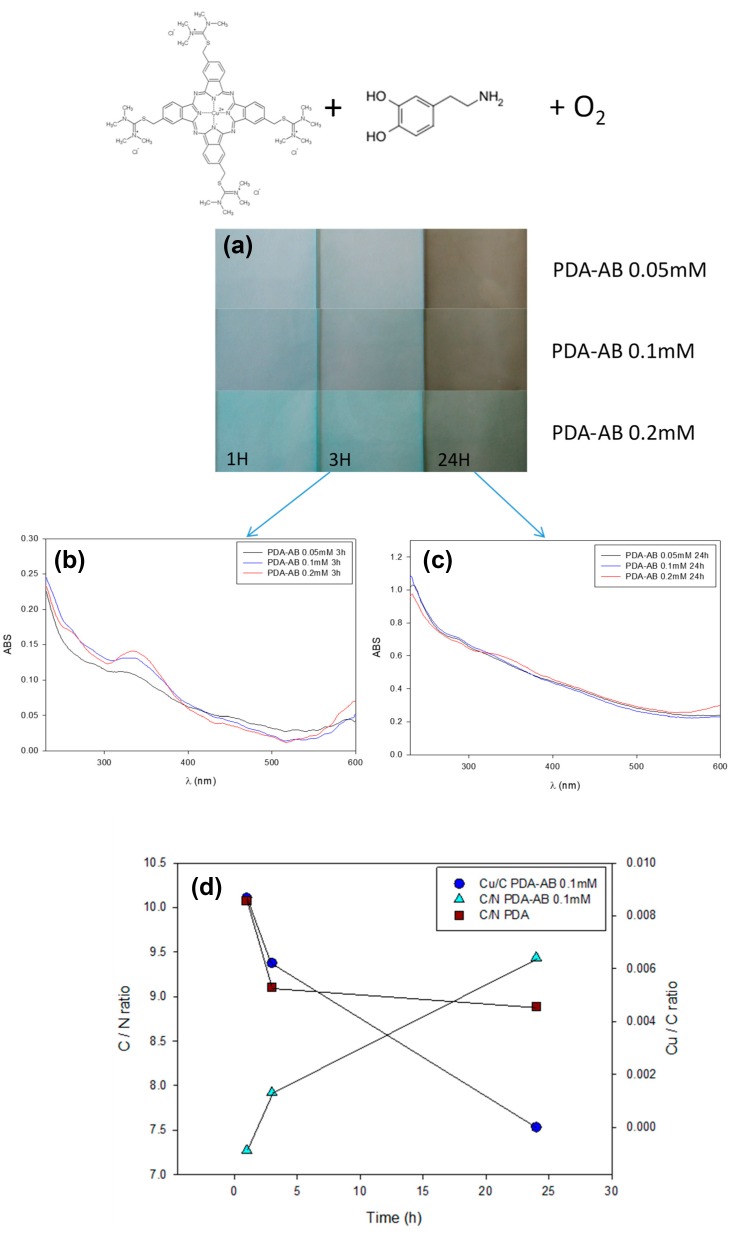
Composites obtained by adding Alcyan Blue (AB) in an oxygenated dopamine solution as shown at the top. (**a**) Digital pictures of glass slides after being put in dopamine–AB blends with AB concentrations of 0.05, 0.1, and 0.2 mM after 1, 3, and 24 h of reaction. The dopamine concentration was the same, 10.6 mM, in all experiments and the films were deposited from 50 mM Tris buffer at pH 8.5 using dissolved O_2_ as the oxidant. (**b**) Ultraviolet-visible (UV-Vis) spectra taken on quartz slides put in dopamine–AB mixtures after 3 h of reaction and increasing the AB concentration from 0.05 to 0.2 mM as indicated in the inset. (**c**) UV-Vis spectra taken on quartz slides put in dopamine–AB mixtures after 24 h of reaction and increasing the AB concentration from 0.05 to 0.2 mM as indicated in the inset. (**d**) Evolution with time of the Cu/C (●) and the C/N (▲) ratios for the polydopamine (PDA)–AB (0.1 mM) films, and the C/N (■) ratio for the PDA films. These atomic ratios are obtained from X-ray photoelectron spectroscopy (XPS). Reprinted from [36], Copyright (2015), with permission from Elsevier.

These strong interactions (of an electrostatic nature and completed by π–π interactions) between porphyrins and phtalocyanines and melanin-like materials have been exploited to incorporate cationic Cu(II) phtalocyanines in PDA films by dissolving Alcyan Blue (AB) up to 0.2 mM in a 10.6 mM dopamine solution at pH 8.5. Dopamine was oxidized by dissolved O_2_ to yield PDA [7]. The obtained films incorporate AB as manifested by a marked blue color and the presence of Cu in the X-ray photoelectron spectra (XPS). This incorporation of dye is apparent after short reaction times, but the spectral signature of AB and the detection of Cu is not possible anymore after longer reaction times, typically after about 10 h (Figure 2) [36]. This decrease of the characteristic optical and compositional signature of AB does not originate from leaching out from the film but from a quantitative incorporation in the composite PDA–AB film: all the available AB (with a solubility in water limited to about 0.3 mM) is incorporated in the film after a short reaction time and when the deposition is continued for longer deposition times, only small oligomers of oxidized dopamine are incorporated in the film to allow for the deposition of almost “pure” PDA. Then the upper parts of the film, close to the film solution/interface reflect the composition of PDA and not of PDA–AB. If homogenous PDA–AB films have to be deposited, the substrate should be exposed regularly to fresh dopamine–AB mixtures [36].

### 3.2. Layer-by-Layer Deposition of Polydopamine-Based Materials

Negatively-charged PDA aggregates and polycations can be assembled into composite thin films using the layer-by-layer deposition method [37,38,39]. The obtained films are more transparent and more permeable for a redox probe like hexacyanoferrate anions, than PDA films directly deposited on the same substrate from a dopamine solution [40] 

The PDA particles obtained by adding poly(allylamine hydrochloride) (PAH) in the reaction mixture are of a controlled size (depending on the dopamine/PAH ratio) and positively-charged. They can be assembled with polyoxometalate anions to yield electroactive films [41]. 

An alternative strategy to incorporate PDA in films deposited according to the layer-by-layer deposition method is to use those films as templates for the formation of PDA from dopamine solutions in the presence of O_2_ as an oxidant [42,43].

**Figure 3:**
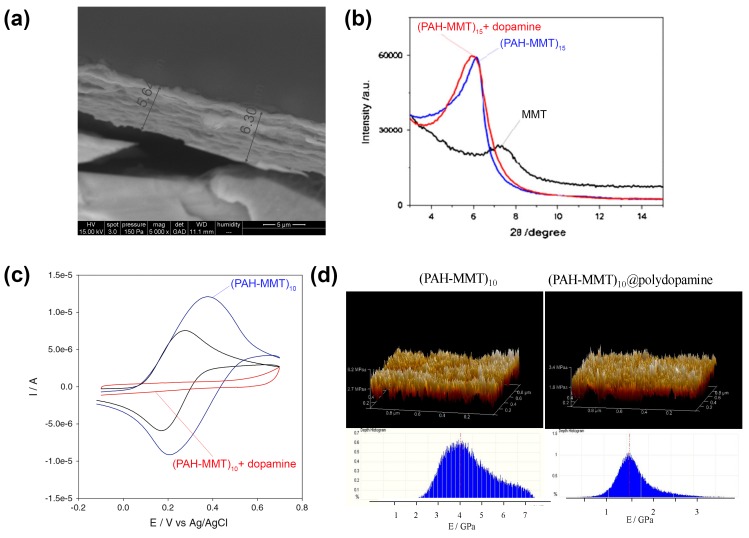
Layer-by-layer deposition of clay and poly(allylamine hydrochloride) (PAH) followed by post-modification with polydopamine. (**a**) Cross-sectional scanning electron microscope (SEM) image of a poly(allylamine hydrochloride)–montmorillonite (PAH-MMT)_15_@polydopamine-coated film obtained after 14 h of contact with dopamine (2 mg mL^−1^) in oxidizing conditions (pH = 8.5, O_2_ as the oxidant). (**b**) X-ray diffractogram of the MMT powder (**^____^**) of (PAH–MMT)_15_ film before (**^____^**) and after (**^____^**) 14 h of contact with dopamine in oxidizing conditions. (**c**) Cyclic voltammetry (at a potential scan rate of 100 mV s^−1^) measured on a pristine amorphous carbon electrode (**^____^**), on the same electrode covered with a (PAH-MMT)_10_–PAH film (**^____^**) and after 14 h of dopamine oxidation (**^____^**). The redox probe was 1 mM K_4_Fe(CN)_6_ dissolved in 50 mM Tris buffer + 150 mM NaCl (pH = 8.5). (**d**) Surface topography over (1 µm × 1 µm) of (PAH–MMT)_10_ and (PAH–MMT)_10_@polydopamine films and distribution of their corresponding elastic moduli. Reproduced with permission from [43].

When dopamine is oxidized in the presence of a polyelectrolyte multilayer film made from the alternated deposition of poly(l-lysine) (PLL) and hyaluronic acid (HA), the film becomes rougher, and homogeneously filled over a thickness of about 1 µm with PDA. The mobility of PLL labeled with fluorescein as a fluorescent probe is reduced (its diffusion coefficient decreases from 5.8 to 3.2 × 10^−2^ µm^2^ s^−1^ after 1 h of dopamine oxidation) and the mechanical properties of the composite film are affected: a (PLL–HA)_24_ film spontaneously dissolves when put in ultrapure water but after only 4 h of contact with dopamine in oxidizing conditions, it can be detached from its quartz support as a free-standing membrane after short immersion in a 0.1 M HCl solution [42]. The same concept has been adapted for films made from the alternate adsorption of poly(allylamine) and clay platelets (montmorillonite, MMT). The obtained composite films kept the layered structure of the original (PAH–MMT)_n_ films (Figure 3a,b), became impermeable to hexacyanoferrate anions (Figure 3c), smoother and more homogeneous in their elasticity even if the average Young’s modulus (1 GPa) decreased with respect to the unmodified multilayered film (4.5 GPa) (Figure 3d) [43].

A multi-layered film made of (3-aminopropyl)trietoxysilane (as an anchoring layer), PDA (as a covalent attachment platform) and stearoyl chloride (as a final lubricating layer) was deposited on glass to produce a robust lubricating composite with a two-fold decreased friction coefficient with respect to glass [44].

### 3.3. Dopamine Grafted on Polymers and Gels

Many researchers have shown the possibility to graft dopamine onto polymers [45,46] and to use these materials to design gels for biomedical applications. Of the highest interest was the demonstration that vanadyl cations (VO^2+^) can gelify chitosan modified with catechol groups at a low vanadyl/catechol ratio, whereas Fe^3+^ cations allow the production of more rigid gels, only at higher cation concentrations. This will allow the production of polymer–catechol composite hydrogels based on metal coordination with a lower cytotoxicity as evaluated with NIH3T3 cells [47].

A mixture of alginate modified with catechol groups and pluronic bis-SH (comporting end-chain functional groups) allows the production of physical gels above the lower critical solution temperature of pluronic bis-SH. These gels can subsequently be cross-linked via catechol and thiol moieties in the presence of a strong oxidant, NaIO_4_. The obtained gels not only display excellent antibacterial activities against *Porphyromonas gingivalis*, but also excellent biocompatibility towards a human line of gingival fibroblasts [48].

### 3.4. Polydopamine–-Protein Composites

**Figure 4:**
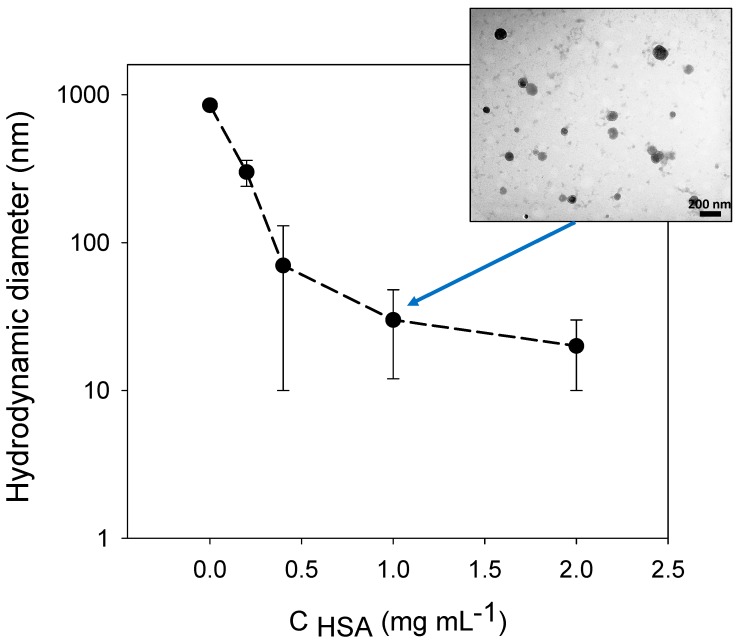
Variation of the hydrodynamic diameter of polydopamine (PDA) particles, as measured by dynamic light scattering; with the concentration of human serum albumin (HSA) added in the dopamine solution (10.6 mM in the presence of 50 mM Tris buffer at pH = 8.5). Dopamine has been oxidized with dissolved O_2_ (open vessel) for 24 h at room temperature before the measurement. Reprinted from [52], Copyright (2014), with permission from Elsevier. The inset (personal data from the author) represents a transmission electron micrograph of the PDA suspension obtained in the presence of HSA at 1 mg mL^−1^.

Inspired by the strong binding of proteins on natural melanin grains [49], and the fact that PDA is an excellent support for protein binding [50] in an active state [51], it was believed that PDA synthesis in solution would be influenced by the presence of proteins. Indeed, it was found that human serum albumin (HSA) resulted in a decrease in the size of PDA aggregates in solution after 24 h of dopamine oxidation in a protein/dopamine ratio-dependent manner (Figure 4). Simultaneously the deposition of PDA on the solid–liquid interface was reduced when the concentration of dissolved HSA increased [52]. 

Similar results have been obtained when dopamine was oxidized in the presence of proteins from chicken egg white [53] suggesting that a large, but yet unknown, set of proteins allows the interaction and interference with PDA during its formation.

### 3.5. Polydopamine–Polymer Composites

When dopamine is oxidized in the presence of poly(vinyl alcohol) (PVA) the obtained polydopamine particles are of controlled size [54] in a manner similar to what has been described in the case of HSA and the proteins from chicken egg white.

PVA is also incorporated in PDA coatings when this polymer is added in the dopamine solution. Surprisingly, when poly(*N*-vinylpyrrolidone) is added to the dopamine solution, no PDA film deposition was found [55]. When poly(*N*-isopropylacrylamide) (PNIPAM) is added to the dopamine solution, the obtained PDA@PNIPAM coatings display temperature-dependant interactions with proteins and cells owing to the presence of the temperature-sensitive polymer in the composite coating [56].

Polymer membranes can be easily functionalized with PDA which allows to confer higher selectivity in filtration processes as reviewed recently [57]. Such composite membranes of high hydrophilicity or hydrophobicity allow for the separation of oil from water [58] 

**Figure 5:**
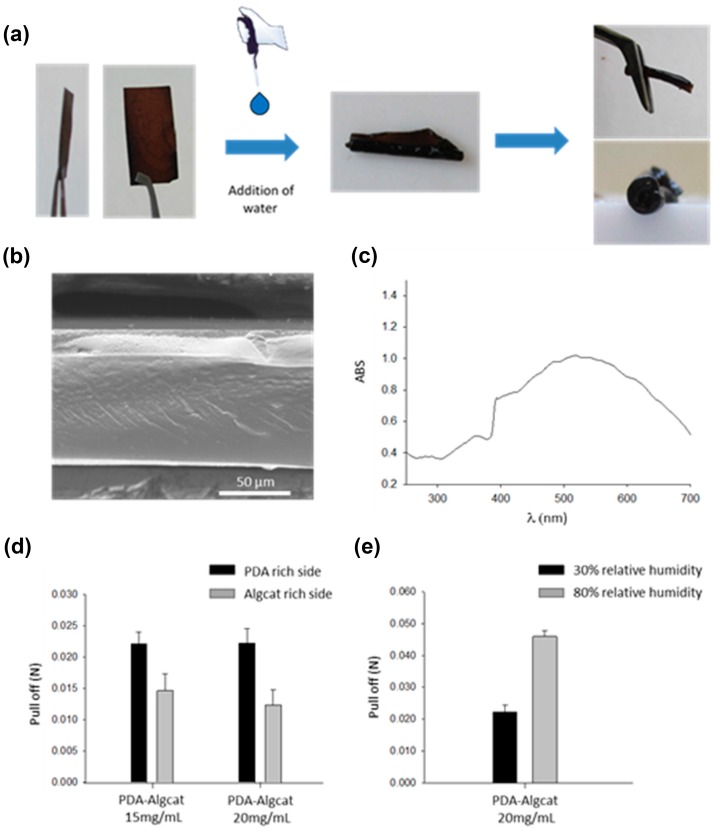
Composite alginate–catechol@polydopamine membranes. (**a**) Images showing the responsivity of PDA-alginate–catechol (PDA-Algcat) membranes to the addition of water. (**b**) Scanning electron microscopy (SEM) images of the dry membrane obtained from an Algcat solution at 20 mg mL^−1^ and (**c**) its UV-Vis spectrum. ABS: Absorbance. (**d**) Pull-off data of a PDA-Algcat membrane at different concentrations of Algcat depending on the side of the membrane (30% relative humidity), and (**e**) pull-off data as a function of the relative humidity. Reprinted from [61]. Copyright (2017) American Chemical Society.

PDA not only forms films at the solid–liquid and liquid–liquid interfaces, but also at the water–air interface. However, the obtained films are apparent only in the absence of agitation because the formed PDA films are too brittle to withstand strong shear stresses [59]. Nevertheless, these materials can be useful because they are transferable to the surface of solids, for instance, poly(tetrafluoroethylene), by means of a horizontal Langmuir–Schaeffer transfer. This offers the interesting opportunity to coat only one face of a planar substrate. However, for many applications, a robust and flexible PDA-based film may be useful. To that aim, it has been shown that the addition of branched poly(ethylene imine) (PEI) to the dopamine solution allows the fabrication of robust free-standing membranes that can be handled with tweezers and which display an anisotropic composition: they are PDA-rich close to the film–air interface, and PEI-rich close to the water–film interface [60]. Later, this concept has been extended with alginate–catechol in the dopamine solution to yield Janus-like free-standing films that change their shape in response to changes in the relative humidity (Figure 5) [61]. The adhesion strength of those membranes are humidity- and side-dependent.

### 3.6. Polydopamine and Conductive Polymers

The electrical conductivity of melanins and PDA films is low, humidity-dependent, and lies typically in the 10^−13^–10^−5^ S cm^−1^ range [62]. This conductivity is most probably of protonic nature, but also implies some redox process due to the composition of melanins [63,64]. The simultaneous presence of hydroquinones, semiquinones (radical species), quinones, and quinone imine forms allow for many kinds of charge storage, hence, allowing interesting applications of melanins and PDA films as supercapacitors [65]. 

In addition, PDA is highly biocompatible, which is not the case for many conductive polymers. The emerging concept is then to try to blend both kinds of materials, PDA and conductive polymers, to find a good compromise between acceptable biocompatibility and improved electrical conductivity. This would allow the design of new flexible electrodes for stimulation of cells, neurons, etc.

PDA has been blended with polyaniline (PANI) in a reactive layer-by-layer manner. PDA was deposited from dopamine solutions at pH 8.5 using O_2_ as the oxidant whereas PANI was deposited from cold acidic solutions using formic acid as the dopant and sodium peroxodisulfate as the oxidant [66]. The obtained layered films displayed a conductivity close to 10^−2^–10^−1^ S cm^−1^ after immersion in a 1.5 M HCl solution. The conductivity of the films decreased when the mass fraction of PDA increased. Nevertheless, this deposition method is cumbersome and of multistep in nature. 

**Figure 6:**
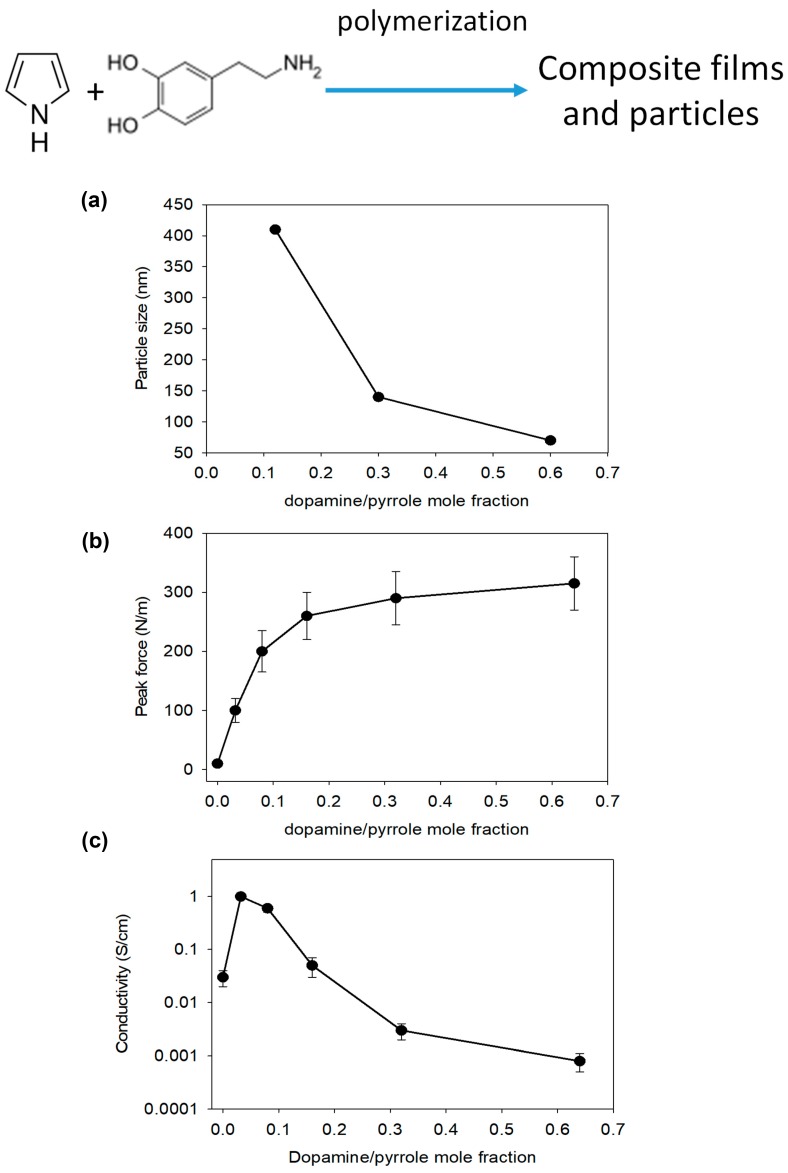
Influence of the presence of dopamine in pyrrole solutions on the properties of the obtained particles. (**a**) Particle size as determined by dynamic light scattering and films; (**b**) Peak force in peeling tests; (**c**) Electrical conductivity after polymerization in the presence of ammonium persulfate. Pyrrole and dopamine structures are shown at the top. Adapted with permission from [68].

The possibility to deposit PDA films from slightly acidic solutions (pH = 5, 50 mM sodium acetate buffer) using strong oxidants (like sodium periodate) [67] will perhaps offer the opportunity to deposit composite PDA–PANI films from solutions containing a mixture of dopamine and aniline. However, it is not excluded that aniline could react with 5,6-dihydroxyindole (the final oxidation state of dopamine) to yield a new compound and a film with different properties. Indeed, when mixtures of dopamine and pyrrole are oxidized with ammonium persulfate, the obtained particles are of smaller size than those obtained in the presence of pyrrole only (Figure 6a), they adhere much more strongly on the surface of solid substrates (Figure 6b), and the electrical conductivity passes through a maximum (of about 1 S cm^−1^) at dopamine/pyrrole mole fraction of about 0.06 [68]. The electrical conductivity of films and pellets of the obtained particles decreased when the synthesis was performed at a higher mole fraction in dopamine because the fraction of insulating PDA increased (Figure 6c). The marked increase in conductivity observed at low mole fraction in dopamine was attributed to better cohesion and adhesion between neighboring particles. Similar results were obtained with l-DOPA or 1,2-dihydroxybenzene added to pyrrole in the polymerization batch. All these results were interpreted on the basis of the formation of a copolymer in which the catechol units are incorporated in the polypyrrole chains.

### 3.7. Polydopamine-Based Composites for Improved Sensing

PDA was deposited in the internal lumen of a 0.5 mm polyetheretherketone (PEEK) tube, a dialdehyde starch porous composite was then fixed on this adhesion coating. The composite coating allowed for the detection of hexanal and 2-butanone derivatized with 2,4-dinitrophenyl hydrazine. The released conjugates where then quantified by means of high-performance liquid chromatography (HPLC). This eco-friendly and cheap conjugate could be used up to 30 times without a notable decrease in performance. The limit of detection for hexanal and 2-butanone as specific markers for liver cancer, was of 1.4 and 1.6 nmol L^−1^, respectively, comparable with other solid phase extraction methods. Statistical analysis showed that this method allowed to distinguish patients with liver cancer from healthy patients [69].

## 4. Future Perspectives of Polydopamine-Based Composites

Even if PDA has shown to be an interesting material to build up composites to yield either core shell nanoparticles, composites with carbon-based nanomaterials, multilayered films, intercalated composites, and stable PDA–protein bioinspired nanoparticles, no doubt that the design principles of such composites will be improved if we gain a better understanding of the structure of PDA. Moreover, the question of a polymeric structure or more probably a self-assembled aggregate of small oligomers of 5,6-dihydroxyindole is still open [70]. If PDA is, indeed, a self-assembled structure with local graphitic order, as suggested by high-resolution transmission electron micrographs [71,72], one may think about processing methods to increase the size of ordered domains in PDA to produce composite materials having a higher electrical conductivity. In this context, defining synthesis conditions allowing to produce a conductive material from a mixture of aniline (yielding to polyaniline in oxidative conditions) and dopamine (or other catecholamines) is challenging. Indeed, PDA is most often obtained in basic conditions, whereas doped polyaniline is ideally obtained in strongly acidic conditions.

The design of PDA-based composites for the design of biomaterials with antibacterial properties and composites allowing for improved adhesion on two different materials will certainly benefit from a better understanding of the structure of PDA and melanin. The ageing process of such composites in real usage conditions has to be investigated in a more detailed manner even if the ageing of PDA in the harsh conditions of proton exchange membranes in fuel cells appears promising [22].

The de-excitation mode of photoexcited melanins and PDA into phonons [73] to produce heat has almost not been exploited in the design of eumelanin- and PDA-based composites.

Finally, the most important recent finding is the possibility to produce stable PDA-based suspensions, in a biomimetic manner, in the presence of proteins in the reaction medium. Such nanoparticles could be processed in two- and three-dimensional printing processes to yield new scaffolds for the design of new biomaterials and materials aimed for energy conversion processes.
